# Exploring Condom Use Among Adult Cisgender Black Women in Texas: a Qualitative Analysis

**DOI:** 10.1007/s40615-026-02971-7

**Published:** 2026-04-21

**Authors:** Amber I. Sophus, Junior Lloyd Allen, Kelsey L. Burton, Déjà Clement, Joyonna Gamble-George, Natalie M. LeBlanc, Jasmine Abrams

**Affiliations:** 1 Department of Health, Behavior, and Society, University of Texas at San Antonio Kate Marmion School Public Health, 8403 Floyd Curl Drive, San Antonio, TX, USA; 2 School of Social Work, Morgan State University, Baltimore, MD, USA; 3 Department of Social Sciences and Health Policy, Wake Forest University School of Medicine, 1 Medical Blvd, Winston-Salem, NC 27104, USA; 4 Chobanian & Avedisian School of Medicine, Department of Psychology, Boston University, Boston, MA, USA; 5 Boston Medical Center, Department of Psychiatry, Boston, MA, USA; 6 Center for Interdisciplinary Research on AIDS, Yale University, New Haven, CT, USA; 7 Environmental Sciences Graduate Program, Oregon State University, Corvalis, OR, USA; 8 University of Rochester School of Nursing, Rochester, NY, USA; 9 Department of Social and Behavioral Sciences, Yale University, New Haven, CT, USA

**Keywords:** Black women, African American women, Condom use behaviors, Health behavior, Sexual risk reduction, Interviews

## Abstract

**Background:**

Cisgender Black women (CBW) remain disproportionately affected by HIV in the United States. Condoms, when used consistently and correctly, are an effective and reliable method for reducing acquisition and transmission of HIV and other sexually transmitted infections (STIs). However, consistent condom use among CBW is mixed. This study aimed to garner a contemporary conceptualization of factors impacting condom use among CBW with male sexual partner(s) residing in large urban areas in Texas.

**Materials and methods:**

Between December 2020 and January 2022, CBW (*N* = 154) were recruited to complete a one-time online survey about HIV prevention and women’s sexual health. Descriptive statistics were used to characterize the study sample by condom use behavior (used condoms/did not use condoms). An inductive qualitative content analysis (IQCA) was used to analyze two open-ended questions about participants reasoning for their condom use or non-use.

**Results:**

Condom use during vaginal sex was similarly proportioned (48.7% users; 51.3% non-users), indicating CBW differed in their condom use behaviors. Four themes described contributing factors for condom use (HIV/STI prevention and family planning) and non-use (relational context and intrapersonal factors). Condom use was motivated primarily by perceived HIV/STI and pregnancy prevention, including dual-protection strategies. Condom non-use was driven largely by relational context, such as trust, perceived monogamy, relationship length, and male partners’ preferences, and intrapersonal factors related to pleasure, comfort, or contraceptive reliance. Across both groups, CBW emphasized partner dynamics, communication, and relationship expectations in shaping decisions.

**Conclusion:**

The qualitative findings highlight how individual, relational, and intrapersonal factors intersect to influence condom use among CBW.

## Introduction

Despite advancements in HIV prevention and treatment services, cisgender Black women (CBW) remain disproportionately affected by HIV in the United States (U.S.). National surveillance data indicate that while CBW comprise approximately 13% of the U.S. female population, they account for over 50% of new HIV diagnoses among all women [1–3]. In the U.S., CBW are 10 times more likely to be diagnosed with HIV compared to White women [1]. The highest rates of HIV among CBW are reported in the southern U.S [4, 5]. Texas is among the states with the highest in HIV prevalence and incidence rates nationally [4] and is home to one of the largest Black populations in the country [6]. Surpassing national disparities, the HIV incidence rate in Texas is 14 times higher among Black women than their White counterparts, and 7 times higher than that of Hispanic women [5, 7]. Since 2012, nearly half of Texas women under the age of 25 who were diagnosed with HIV have been CBW [1, 2, 8]. These disproportionate rates of HIV incidence among CBW highlight a critical public health issue.

Given that the most common mode of HIV transmission among CBW is heterosexual contact [5, 8–10], condoms remain one of the primary methods of prevention. When used consistently and correctly, male and female condoms are effective and reliable at reducing acquisition and transmission of HIV and other sexually transmitted infections (STIs) [11, 12]. The benefits of condoms extend beyond individual protection, helping to reduce community-level transmission by disrupting sexual networks [11, 12]. However, prior research on condom use among CBW has been mixed. Some studies have found higher condom use among partners of Black women compared to White or Hispanic women [13–16], whereas others have reported low levels of consistent condom use among CBW [17]. While condom use rates may be higher among CBW in some contexts, it’s important to consider these discrepancies and understand the various factors that influence CBW’s condom use decisions and ability to negotiate condom use with male partner(s) so that interventions can be appropriately tailored.

Although many CBW report condomless sex within relationships they perceive as committed or monogamous, relationship status alone does not fully determine HIV/STI risk or vulnerability. Women’s susceptibility can be shaped by factors outside their control, including partners’ sexual histories, undisclosed concurrency, untreated sexually transmitted infection(s), and broader contextual conditions [18–20]. Thus, understanding condom use within heterosexual relational contexts remains critical, even among women who view their relationships with male partners as stable or exclusive. These complexities underscore the need to examine CBW condom use communication and subsequent decision-making across different relationship types and relational contexts. For instance, condom use among women in long-term heterosexual relationships matters not because it is unusual, but because it reveals important dynamics related to health, trust, and power that directly affect women’s well-being. Although such relationships are often assumed to be low risk, STIs and unintended pregnancies can still occur, and women tend to bear the greater biological and social consequences.

Building on this relational framing, a range of intrapersonal and relational factors further shape CBW’s condom use decisions. Despite the effectiveness of condoms, correct and consistent use is significantly shaped by relationship type (e.g., married versus casual), sexual pleasure [21], condom negotiation self-efficacy [22–24], and relationship context and dynamics. For instance, the fit and feel of condoms (i.e. latex allergy, decrease friction) for men or women, as well as concerns about decreased sexual pleasure, affect condom use [21]. Previous research has also shown that, at the individual level, self-efficacy in negotiating and communicating with sexual partner(s) about condom use is a strong predictor of consistent condom use [22–25]. Women with high levels of self-efficacy are more likely to use condoms consistently compared to women who have less self-efficacy [22–25]. Moreover, the decision to use condoms often hinges on the male partner’s comfort, influence, and the relationship type and dynamics. Several studies have identified that relationship context influences condom use behaviors, such that condoms are less likely to be used in relationships with assumed monogamy, while used more frequently in new and casual relationships [26–30].

Relationship dynamics such as trust further dictate condom use. Trust has been identified as a determining factor in women’s ability to negotiate safer sex and likelihood to engage in condomless sex [18, 31–34]. Moreover, relationship expectations frequently frame condom discontinuation as a sign of trust or commitment, which can discourage use even when condom use may be warranted. At the same time, women may have limited power to negotiate condom use due to partner resistance or fear of conflict, and responsibility for contraception is often shifted onto women through female-controlled methods that do not protect against STIs. Perceived gendered power imbalances within sexual relationships can also limit CBW’s advocacy for condom use and reduce their self-efficacy in negotiating condom use [35, 36]. These imbalances are often rooted in gendered social norms that position men as the primary decision-makers in sexual relationships. CBW in such relationships may feel compelled to prioritize their male partner’s desires over their own sexual health [22, 24, 37, 38]. They may also tolerate or overlook non-monogamy and concurrent sexual relationships [35, 38], especially if there is a perceived high level of commitment and familiarity with the partner. In situations where CBW are financially dependent on their partner or experiencing intimate partner violence, they may avoid initiating conversations about condom use to prevent conflict or avoid being perceived as unfaithful, further discouraging condom negotiation [35, 37].

Although numerous studies have examined HIV risk and prevention among CBW, important gaps remain. First, much of the existing HIV prevention research focused on CBW centers on younger populations, often overlooking the experiences of those who are middle-aged or older. Second, there is limited research that captures the perspectives of CBW in states like Texas, where HIV rates and related vulnerabilities remain disproportionately high. Third, while CBW may possess knowledge about HIV and the benefits of condom use, knowledge alone does not always translate into consistent condom use [22, 24, 39], highlighting the need to explore how CBW are navigating condom use decision making in the context of various relationship types. This question is especially timely and merits an up-to-date investigation given medical and bio-behavioral advances in HIV prevention over the past decade (i.e., biomedical prevention methods). Thus, the current study provides a contemporary examination of condom use behaviors among a sample of adult, HIV-negative CBW living in Texas who had engaged in at least one HIV risk behavior in the past six months. We aimed to answer the following research question: What factors influence cisgender Black women’s condom use behavior with their male sexual partner(s)?

## Methods

### Procedures and Eligibility Criteria

Between December 2020 and January 2022, CBW were recruited from Houston, San Antonio, and Dallas, Texas (all designated Ending the HIV Epidemic priority jurisdictions [40]) to complete a one-time online survey about HIV prevention and women’s sexual health [41]. Participants were recruited using online advertisements and print flyers that contained information about the study and a weblink to access the online consent form and eligibility screener. Online advertisements were placed on Facebook and Instagram (clickable weblink embedded), whereas printed flyers (with a QR code and weblink) were placed in locations in Houston, Dallas, and San Antonio frequented by Black women (e.g., coffee shops, restaurants, grocery stores, gyms) [42].

Irrespective of recruitment method, all interested individuals were taken to the study landing webpage housed in Qualtrics, a HIPAA-compliant survey program (Qualtrics, Provo, UT). The study landing webpage provided information about the study, the electronic consent form, and the online eligibility screener. Respondents who consented to participate in the study were immediately routed to the eligibility screener. For study inclusion, participants had to: (1) be at least 18 years old; (2) self-identified as a cisgender woman; (3) self-identified as Black (e.g., African American, Caribbean American [e.g., Haitian American]); (4) have an HIV-negative or unknown serostatus; (5) live in or near (within 25 miles) Houston, San Antonio, or Dallas, Texas; (6) have engaged in at least one Centers for Disease Control and Prevention (CDC)-defined HIV risk behavior within the past 6 months (i.e., engaged in condomless vaginal and/or anal sex with male partner; injection drug use; sex exchange; and/or had a sexually transmitted infection [STI]); and (7) be fluent in English. The behavioral eligibility criteria used in this study therefore reflects the National Institutes of Health (NIH) and the Joint United Nations Programme on HIV/AIDS (UNAIDS) defined indicators of HIV exposure, of which classify condomless vaginal or anal sex (especially with a partner whose HIV status is unknown), injection drug use, recent STI diagnosis, and sex exchange as HIV risk behaviors that increase measurable HIV acquisition risk [43–45]. Consistent with UNAIDS guidance, we distinguish HIV “risk” (behavioral exposures) from HIV “vulnerability.” Vulnerability encompasses structural, interpersonal, and contextual conditions, such as partner concurrency, gendered power dynamics, limited access to prevention resources, and community-level HIV prevalence, that are beyond an individual’s control and may heighten susceptibility to HIV infection even when individual behavior appears low-risk. HIV vulnerability may also include intrapersonal factors, such as limited knowledge, skills, or self-efficacy for HIV prevention, that are shaped by broader structural or interpersonal conditions and reduce an individual’s ability to avoid HIV risk [43].

Individuals who provided consent, and were eligible, completed the one-time 30-minute web-based survey via Qualtrics (Qualtrics, Provo, UT). Enrolled participants (those who provided consent, were eligible, and completed at least 80% of the survey) were emailed a $25 USD electronic gift card for their time. The total enrolled sample included 154 participants. Detailed enrollment procedures have been previously reported [41]. The Institutional Review Board at the University of Hawai’i at Mānoa approved all study procedures.

### Measures

#### Demographics

The online survey contained closed and open-ended free-text questions. The current study focuses on closed and open-ended questions related to condom use. To obtain sample characteristics, participant data was captured on age, Hispanic ethnicity, employment status, education, household income, family planning provider, sexual orientation, current engagement in vaginal sex with a male sexual partner, and relationship status. Women who indicated being in a relationship were asked about their relationship type and relationship length. Women self-reported if they engaged in at least one of the following HIV risk behaviors within the past 6 months prior to study participation in the eligibility screener: condomless vaginal sex with a male, condomless anal sex with male partner; use of illicit drugs by needle injection; sex exchange; and/or had a sexually transmitted infection [STI]. The sex exchange survey item did not assess what was exchanged (e.g., money, housing, goods), which restricts our ability to characterize the context of sex exchange as it relates to HIV risk and vulnerability. Participants were also asked if they had ever tested for STIs (other than HIV) in their lifetime, and about their HIV testing history in the past 6 months.

#### Condom Use

To quantitatively examine condom use behaviors, women were asked about their frequency of using condoms during vaginal sex in the past 6 months, with categorical response options: ‘Never,’ ‘Sometimes,’ and ‘Always’ [46]. We examined descriptive characteristics across the three original categories to ensure that “sometimes” and “always” users were not distinct subgroups for the purposes of contextual description. Because the purpose of this study was to capture whether participant’s engaged in any condom use with a male sexual partner rather than to compare behavioral frequency patterns, condom use was recoded into a dichotomous variable; such that ‘condom use’ included participants who responded “always” and “sometimes,” and ‘non-condom use’ included participants who responded “never.” We refer to “users” as those who did use condoms during vaginal sex with a male within the past 6 months (either sometimes or always) prior to study participation. This approach is consistent with prior HIV prevention research that operationalizes condom use as a binary indicator [22, 47, 48]. Women were also asked if they had ever discussed condom use with a sexual partner (binary response: yes/no), and if yes, who first brought up the topic of using condoms (‘My partner’, ‘Me’, ‘I don’t remember’). Participants were then asked if they wanted to use condoms (binary response: yes/no), and if their partner wanted to use condoms (binary response: yes/no).

#### Qualitative Assessment of Condom Use Behaviors

Women were asked two open-ended survey questions to qualitatively explore why they did or did not use condoms in the preceding six months. To anchor their responses to a consistent timeframe, these questions were introduced after asking about their condom use with a sexual partner (vaginal or anal sex) during the past six months, ensuring that participants had a clear timeline in mind. The open-ended questions were: “What was your reason for using condoms when you had sex?” and “What was your reason for not using condoms when you had sex?” These questions asked about participants’ general experiences over the past six months but were not tied to any specific partner type (e.g., main vs. casual partners). Our approach in this research prioritized insights generated from these qualitative findings.

### Data Analysis

Descriptive statistics (mean, standard deviation, and frequencies) were calculated to characterize the study sample and summarize all variables by condom use behavior. All statistical analyses were conducted using statistical software R, version 3.4.1 (The R Foundation). A qualitative descriptive approach was used for the open-ended responses. An inductive qualitative content analysis (IQCA) was used to analyze qualitative data [49–51]. IQCA uses descriptive methods for coding, interpretation, and reporting [52, 53] and involves using systematic coding and categorization strategies to identify trends, patterns, word frequencies, relationships, and communication structures [49–51]. Completing the IQCA approach involved three phases: preparation, organization, and reporting [54]. The preparation phase in IQCA involves setting the foundation for the study. In this phase, the research team defined the study purpose, unit of analysis, and its focus. For this study, the unit of analysis were the two open-ended survey questions regarding condom use.

First, the data was organized by each open-ended question. One researcher (JLA) initially drafted a coding mechanism, steps to support reliability, and checks between coders. The next phase, organization, included systematically coding the data into meaningful categories. In this stage of analysis, three team members (JLA, AIS, KB) reviewed the qualitative data stemming from the two open-ended questions and used the coding mechanism to employ an open coding process to identify emerging codes. As new insights into the data emerged, the coding process was further refined. The codes were then grouped together to create themes that captured their shared meaning. Once all three team members completed this process individually, the team convened for a collaborative analysis session to discuss and refine emerging themes, particularly where themes were unclear or overlapping. After the initial themes were developed and clarified, the research team engaged in multiple rounds of constant comparison, revisiting the data to ensure that the themes were coherent and comprehensive; and checking that supporting quotes were linked. This allowed for a deeper understanding of how individual data is connected to the broader themes. Additionally, the research team checked for any discrepancies and ensured that the themes were truly reflective of the participants’ experiences. As a final step, peer debriefing with team members (JLA, AIS, KB, DC) and two external researchers who are experts in the field (NL and JA) was conducted to further analyze the themes to ensure they were reflective of the qualitative data, and to enhance the credibility of the findings by incorporating diverse perspectives and minimizing potential researcher bias.

The final phase, reporting, ensured that findings were contextualized and reported clearly and systematically. As such, the final qualitative findings were organized around the themes that emerged from this iterative process, ensuring that each was grounded in the data and represented the participants’ perspectives. Tables were created to describe the themes that emerged related to CBW’s condom use behavior (i.e., used condoms or did not use condoms), including theme definitions and supporting quotes. A Venn-diagram was created to highlight the relationships between the themes as they relate to condom use, in general, among the sample. Overall, this systematic approach in analyzing qualitative data allowed the research team to capture nuanced insights into the condom use decisions made by CBW in this study.

### Researcher Reflexivity

The primary data analysis team for this study was comprised of three Black women and one Black man, with backgrounds in Public Health (AS, KLB, DC), Clinical Psychology (DC), and Social Work (JLA). All team members share a deep commitment to understanding health outcomes and behavioral practices that can reduce new HIV infections, particularly within the Black community. Each team member played a critical role in the data analysis phase, interpretation of study findings, and writing of this manuscript, bringing their unique perspectives and expertise to ensure a comprehensive and culturally relevant approach to the interpretation and presentation of the study findings. As Black researchers, we recognized the importance of examining our own unique identities (e.g., sexual orientation, cisgender identity, ethnic background, etc.), lived experiences, and biases, particularly regarding our connection to the communities we are studying. Throughout the research process, we engaged in regular reflection through virtual meetings to critically assess how our identities and positions within the community might shape our interactions with the data, the questions we posed, and the interpretations we made. This process of reflexivity helped us ensure that the analyses and interpretation of the study findings were not only informed by the evidence but also by a deep understanding of the cultural, historical, and social contexts in which these findings exist.

## Results

### Demographic Characteristics

The study sample consisted of 154 CBW, with 79 individuals (51.3%) reporting *not* using condoms and 75 individuals (48.7%) reporting using condoms during vaginal sex with a male partner in the past 6 months prior to study participation (see Table 1). The average age of condom users was younger (29.8 years) compared to non-users (36.8 years), with a larger proportion of condom users in the 18–24 age group (28.0% vs. 7.6%). Non-users were more likely to be aged 35 and above. Across both groups (users and non-users), most participants were non-Hispanic (*n* = 144, 93.5%), employed (*n* = 113, 73.4%), had a bachelor’s degree or higher (*n* = 90, 58.4%), had an income above $35k USD (*n* = 113, 73.4%), were heterosexual (*n* = 134, 87.0%), and were sexually active with a male partner at time of study participation (*n* = 138, 89.6%). A little over half of the participants had a family planning provider (*n* = 89, 57.8%), with more non-users (*n* = 51, 54.4%) having a family planning provider than condom users (*n* = 38, 50.7%). More than half of the participants were in a relationship (*n* = 98, 63.6%), with a higher proportion of single women reporting using condoms (53.3%) than non-users (20.3%). By contrast, most non-users were in a relationship (79.7% vs. 46.7% among users). Among those in a relationship (*n* = 98, 63.6%), across both users and non-users, most were in a monogamous relationship where they only have sex with one another and no one else. A higher percentage of non-users (*n* = 57, 72.2%) had been in their relationship for at least 1 year or more compared to users (*n* = 25, 33.4%).

### Personal HIV Risk Factors

Nearly all participants engaged in condomless vaginal sex in the past 6 months (*n* = 153, 99.3%). A smaller proportion reported engaging in other HIV risk behaviors, such as condomless anal sex (*n* = 26, 17.1%) and sex exchange (*n* = 12, 7.8%). Although these behaviors occurred among both condom users and non-users, they were more frequently reported by participants in the condom use group. This distribution likely reflects underlying demographic and relational differences between groups rather than implying that condom users engage in HIV risk behavior because they use condoms [55]. Most condom users had been tested for an STI (other than HIV) at least once in their lifetime (*n* = 60, 75.9%) compared to non-users (*n* = 44, 58.7%). Among the participants who tested at least once in their lifetime, a higher proportion of condom users had been tested for HIV at least once in the prior 12 months (*n* = 47, 59.5%) compared to non-users (*n* = 25, 33.3%).

### Condom Use Behaviors

Study findings revealed distinct patterns of sexual communication about condoms, with most participants (*n* = 126, 81.8%) reporting having discussions about condom use with their sexual partner(s). However, a higher proportion of condom users initiated these conversations (66.7% versus 41.8%). Additionally, 14.9% (*n* = 23) of participants reported that they did not remember who first brought up the topic. Under two-thirds of participants (*n* = 102, 66.2%) reported wanting to use condoms, while only 37.7% (*n* = 58) reported that their partner shared this preference.

**Fig. 1:**
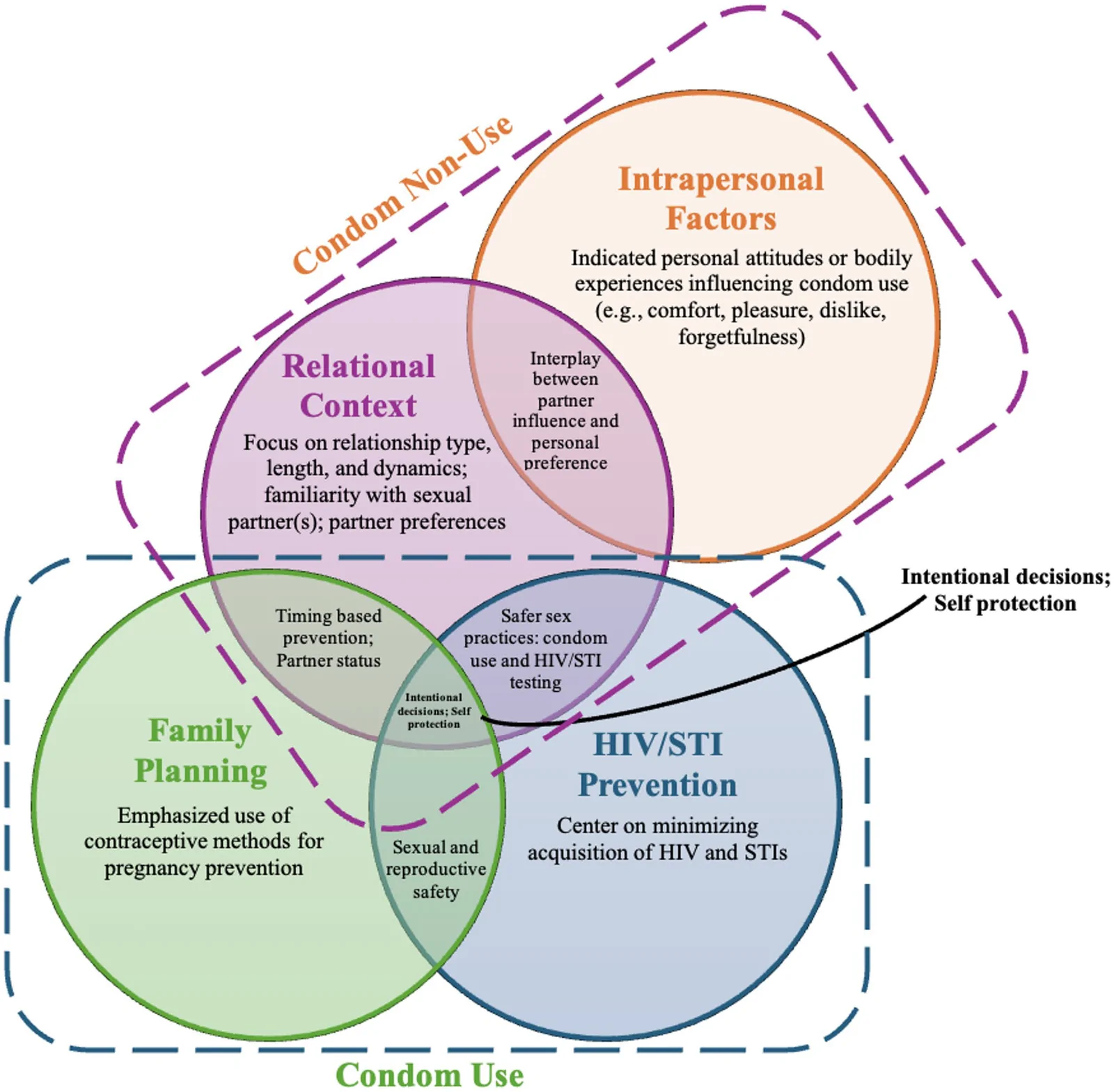
Primary themes associated with condom use behavior among cisgender black women

Two-open ended questions were used to further explore the factors influencing condom use or non-use in this sample. Notably, the same themes emerged in participants’ responses for both condom use and non-use; however, these themes manifested in distinct ways depending on individual experiences. Below, we describe the primary themes associated with women’s decision to use and not use condoms, as well as how these themes intersect with respect to condom use behavior, in general (see Fig. 1).

The Venn diagram illustrates the four primary qualitative themes influencing cisgender Black women’s condom use behavior. “HIV/STI Prevention” and “Family Planning” represent the most frequently reported reasons for condom use. “Relational Context” and “Intrapersonal Factors” reflect the most frequently endorsed reasons for condom non-use. Only themes that co-occurred in participants’ explanations are shown as overlapping. “Contextual factors,” such as condom availabilty and personal preference, emerged as a secondary theme and is therefore not included in this primary theme diagram.

### Factors Influencing Women’s Condom Use

Four themes emerged to explain women’s use of condoms with a male sexual partner: relational context, HIV/STI prevention, family planning, and contextual factors. Of these four themes, HIV/STI prevention (reported by 39 individuals) and family planning (reported by 36 individuals) were the primary reasons why women in this sample chose to use condoms during vaginal sex, whereas relational context (reported by 10 individuals) and contextual factors (reported by 6 individuals) were less frequently mentioned and, therefore, presented as secondary reasons (Table 2).

#### Relational Context

A small subset of CBW indicated that their decision to use condoms was shaped by the nature of their relationship or familiarity with their sexual partner. CBW described using condoms when they were with a “new partner,” “someone [they] did not know” well, or in situations where the partner’s sexual history or the level of trust or commitment was unclear. These relational contexts prompted greater caution and motivated condom use for added protection among the CBW.

#### HIV/STI Prevention

HIV/STI prevention as a primary motivation for their condom use, specifically protecting oneself from HIV and STIs. Participants explicitly stated that they used condoms because they wanted to “avoid getting infected with [STDs, STIs, or HIV].” Others mentioned that they wanted to protect themselves “from being infected with sexually transmitted diseases” and to practice “safe sex.” These statements highlight awareness of risk and vulnerability to HIV acquisition and other STIs and the personal motivation to take preventative measures. One participant shared her experience: “When I had sex for the first time, we forgot to put a condom on. The second time we had sex, we used a condom because we forgot the first time and were taught to have safe sex.” The participant’s reference to being “taught to have safe sex” indicates that education around HIV and STI prevention can influence sexual behaviors, especially in situations where individuals might otherwise overlook using condoms.

#### Family Planning

In relation to family planning, participants described the use of contraceptive methods (i.e., condoms and birth control) to primarily prevent unintended pregnancies. Participants noted that they used condoms because they “did not want to become pregnant anytime soon.” Others shared this type of decision with their partner. For example, one participant stated, “at the time, we did not want kids. Basically, we were just avoiding [getting] pregnant.” The importance of condom use to navigate family planning and protection of sexual health considering infidelity was also highlighted. Someone shared, “[I wanted] to reduce risk of getting pregnant and contracting STD since I had sex with someone who was not my partner.”

For others, condoms were used in conjunction with birth control as an extra step to prevent pregnancy. Among condom users, 36 participants cited pregnancy prevention as their primary motivation, with some employing dual protection strategies that used condoms as backup contraception on fertile days or in conjunction with hormonal methods. One participant who was 39 years old, married, and engaged in condomless vaginal sex reported that condoms were used because, “We do not want any more kids right now, so we also use condoms on my fertile days as a backup to my birth control pills.” Another participant who was 24 years old, dating multiple people, and engaged in condomless anal and vaginal sex reported, “To avoid getting pregnant, especially when it was my ovulation day. It’s much safer to use a condom rather than a birth control pill.” These statements reflect how contraception is not only a measure to prevent unintended pregnancy, but also a tool for managing the timing of childbearing according to individuals’ life goals or readiness for parenthood.

#### Contextual Factors

A small number of CBW described using condoms due to immediate situational factors, such as condom availability or a moment-based “personal decision.” These responses reflected contextual circumstances rather than sustained motivations for condom use and were less frequently endorsed than other themes.

### Factors Influencing Women’s Non-Use of Condoms

Four themes emerged to explain CBW’s reasoning towards not using condoms during sex with a male partner in the past 6 months: relational context, HIV/STI prevention, family planning, and intrapersonal factors. Among these four themes, relational context (reported by 65 individuals), intrapersonal factors (reported by 22 individuals) were the primary reasons why women chose not to use condoms during sex. In contrast, HIV/STI prevention (reported by 3 individuals) and family planning (reported by 10 individuals) were less commonly endorsed and are, therefore, included as secondary considerations for condom non-use (Table 3).

#### Relational Context

Women’s decision to not use condoms with their sexual partner was shaped by the nature of their relationship with their sexual partner(s). This theme encapsulates how relationship type, dynamics (i.e., trust), length, familiarity with a sexual partner, and partner preferences influenced women’s decisions not to use condoms. For some participants, the decision to not use condoms was based on their personal perception of trust and exclusivity within their relationship. As one participant shared, “We are married and we trust each other,” indicating that commitment and trust is inherent in a marital relationship and subsequently diminishes the perceived need for condom use. Similarly, other participants highlighted the monogamous nature of their relationships, with quotes like “We are exclusive,” “We both agreed to be monogamous,” and “We are married and only have sex with each and no one else.” These statements reflect the belief that if both partners are exclusively involved with each other, their risk to acquiring HIV or STIs is minimized, making condom use seem unnecessary.

Additionally, relationship length and familiarity with a sexual partner appeared to play a key role in women’s condom use. Statements like “[I have] been with the same man for 6 years” or “[I’ve had the] same partner for 30 years” revealed that the longer the duration of the relationship, the less likely participants felt the need for condoms. To a lesser extent, some participants, recognizing their partner’s reluctance or preference, chose not to use condoms. For example, some shared “My partner doesn’t like them” or “My partner didn’t want to use.” While this finding does indicate that some women may be prioritizing their partner’s preferences about condoms over their own, this finding could also be reflective of the role differential power dynamics or lack of agency plays within sexual/intimate relationships.

#### HIV/STI Prevention

Only a few CBW attributed their non-use of condoms to routine HIV/STI testing practices with their partners. These women explained that mutual testing reduced their perceived need for condoms, with comments such as “we both test for STD and HIV regularly together” and “we have both been tested,” reflecting a belief that regular screening offered adequate reassurance and protection.

#### Family Planning

Some CBW described not using condoms because their priority was family planning, which was addressed through other means, such as hormonal contraception, tubal ligation, or an active desire to become pregnant. Examples included statements, such as “I’m on birth control,” “my tubes are tied,” and “we are currently expecting.”

#### Intrapersonal Factors

Within the theme of intrapersonal factors, women described their personal attitudes toward condom use, including concerns that condoms may lessen sexual pleasure or increase physical discomfort during vaginal sex. Participant statements, such as “Pleasure,” “I did not want to feel a condom,” “I don’t like them,” “Uncomfortable,” and “I do not enjoy them” suggest how concerns about reduced pleasure or physical discomfort may be significant factors influencing condom use among some women. Moreover, situational elements, such as forgetfulness, although limited, also emerged as another factor that contributed to lapses in condom use. A few women admitted, “At times I forgot [to use condoms].” Another participant acknowledged, “At times I could be so drunk that we forget to use a condom.”

## Discussion

It is well-evidenced that condoms are effective at preventing HIV/STI transmission among CBW, when they are used consistently and correctly [35, 56]; however, decades of research have demonstrated that condom use communication and decisions among Black women are shaped by intrapersonal, relational, and structural factors [18, 56–60]. Seminal research on relationship dynamics, condom negotiation, trust, and gendered power imbalances has repeatedly shown that condom use declines within primary or long-term partnerships and that women’s ability to negotiate condom use is influenced by their partners and broader sociocultural conditions [47, 48, 59–61]. Situating our findings within this established literature provides important context for understanding CBW’s current experiences.

Given the emergence and increased use of new HIV/STI prevention strategies, inclusive of biomedical HIV/STI prevention methods, the current study conducted a contemporary examination to understand factors impacting condom use decisions among CBW residing in large urban areas in Texas. Using IQCA [52, 53], themes emerged to describe factors influencing condom use and non-condom use: HIV/STI prevention, family planning, relational context, and intrapersonal factors. The findings provide additional insights into condom use decision-making among adult CBW beyond adolescent-centered research, and provide an in-depth examination of relational context, contraceptive multiplexity, and pleasure-related considerations that have received limited attention in recent sexual health research with CBW. However, these results do not represent a shift, break, or new direction in condom use behavior, but instead mirror patterns that have been well documented throughout the HIV epidemic. This represents a broader issue in our efforts to reduce HIV/STI incidence rates among CBW and highlights the importance of improving the availability of relationally focused and couple-centered HIV/STI prevention approaches that incorporate trust-building, communication, and dyadic decision-making.

Additionally, these findings hold particular importance for sexual health interventionists and behavior change theorists by emphasizing the need for multi-level, interpersonal, and relationally grounded prevention approaches tailored to CBW’s lived relational and sociocultural contexts. While education remains an important component of sexual health promotion, education alone is insufficient to significantly change condom use behaviors among CBW. Effective HIV/STI prevention requires strategies that address relational dynamics, gendered power, and male partner influence, as well as structural and provider-level factors. These multi-level considerations accentuate the need for interventions that actively engage male partners, strengthen communication within couples, and challenge coercive or dismissive partner behaviors that limit women’s condom use agency.

### The Nature of Relational Context in Condom Use Decision Making

Findings highlight that CBW’s sexual and intimate relationships, and the context of those relationships (i.e., relationship status, type, length, dynamics, and familiarity with one’s sexual partner(s)) play a role in women’s condom use decisions. Similar findings have been identified in prior research, such that, intimate partners, relationship types [19, 27, 62, 63], and relationship dynamics [57, 64] influence women’s susceptibility to HIV and other STIs. Many CBW in this study reported being in long-term, committed, monogamous relationships. These partnerships may increase HIV susceptibility when partners engage in nondisclosed extra-dyadic sexual behavior and/or there is an absence of explicit sexual communication or a sexual agreement about the relationship type (e.g., exclusive or open). Research with Black women across diverse settings demonstrates that undisclosed infidelity and partner concurrency are not uncommon and can expose women to HIV, even when they themselves are monogamous [18–20, 65]. Likewise, qualitative studies show that women often rely on relational trust as emotional reassurance rather than as a verified indicator of physical safety, making it difficult to assess actual “risk” when partners’ behaviors [18, 20, 66] and HIV/STI status are unknown. In the current study, trust was the most cited reason for women’s non-use of condoms (“We are married and we trust each other,” “We are exclusive,” or “We only have sex with each other”). The literature suggests that trust can affect women’s participation in and decision-making surrounding HIV/STI prevention [58–60, 67], inclusive of condom use.

Moreover, many people in long term, committed relationships have an expectation of “safety”. Some women may view being in a relationship, particularly one they perceive as monogamous, trustworthy, and stable, as protective against HIV/STIs, thereby reducing their perceived need for condom use [60, 68–72]. On the other hand, single women may be more perceptive or aware of their potential risk and vulnerability for acquiring HIV and other STIs, and as such, may be more inclined to plan to use condoms. Altogether, as partners may come to equate closeness, trust, and intimacy with safety, this may reduce their concern around HIV/STI prevention [19, 66], and thus reduce their need for condoms to exclusively “protect” against HIV/STIs. These relational uncertainties intersect with structural factors, such as higher community HIV prevalence, limited access to HIV/STI testing, and social expectations surrounding fidelity and condom use, which further elevate HIV vulnerability among Black women, even those in relationships perceived as stable and exclusive [65, 73–76]. These epidemiologic, relational, and contextual factors help explain why monogamy does not necessarily confer protection, and why condomless sex within presumed monogamous partnerships may still place some women at heightened HIV risk. These findings are highly consistent with longstanding sexual health research documenting that condom use typically decreases as emotional intimacy and relationship duration increases [47, 48, 61]. Moreover, women’s ability to negotiate condom use is shaped by gendered power dynamics within heterosexual relationships, a framework articulated within the Theory of Gender and Power and supported by subsequent studies of Black women’s sexual health decision-making [47, 48, 61]. Our findings therefore confirm and extend this foundational literature in a contemporary, post-PrEP context and within Texas-specific sociocultural conditions.

Given that sexual health decisions are dyadic, requiring participation from both partners, these findings suggest that couples-centered approaches [77–79] may be beneficial to improve women’s sexual health and thus improve condom use behaviors. Couples-centered approaches should strive to leverage trust, either by reframing condom use as mutually protective and supportive, or by emphasizing the importance of open communication about HIV/STI status and risk or vulnerability behaviors. Moreover, relational-centered sexual health education that incorporates relational contexts into HIV/STI prevention messaging [80] may be an ideal way to inform individuals of vulnerabilities for acquiring HIV/STIs within the context of their sexual relationships, regardless of relationship type (romantic, nonromantic, transactional, etc.). Although our open-ended survey items did not require participants to specify whether condom use or non-use occurred with main, casual, or non-monogamous partners, many women voluntarily described their relationship contexts in detail (e.g., monogamy, trust, relationship length, partner preferences), providing sufficient contextual grounding for interpreting these findings. Thus, our dyadic and relationship-focused recommendations are directly informed by the relational context participants provided, even without a partner-type–specific prompt.

### Contraceptive Multiplexity and Reproductive Autonomy

Family planning considerations also influenced condom use, such that HIV/STI prevention and pregnancy prevention were primary, often overlapping motivators for condom use. This approach demonstrates reproductive agency and risk management sophistication that merits recognition in clinical and educational settings. Recognizing this agency means acknowledging and validating women’s proactive efforts to balance multiple sexual health risks and make informed choices tailored to their unique needs and circumstances. In clinical practice, this could involve healthcare providers explicitly discussing and supporting comprehensive sexual health strategies that address both HIV/STI prevention and contraceptive needs simultaneously, ensuring discussions are nonjudgmental, individualized, and patient-centered.

Conversely, reliance on other contraceptive methods or medical conditions contributed to condom non-use among some participants, as noted in Table 3. CBW cited hormonal birth control, permanent sterilization procedures, and medical conditions like PCOS as reasons for forgoing condoms. While these approaches address pregnancy prevention, they leave women susceptible to HIV/STI acquisition. This contraceptive substitution pattern reflects a common challenge in sexual health promotion: helping women understand that different contraceptive methods address different risks and that dual protection strategies may be necessary for comprehensive sexual health. The integration of family planning and HIV/STI prevention services is critical for addressing this disconnect, also providing a point of intervention among women who may be more concerned about pregnancy prevention vs. HIV/STI acquisition. This illustrates an opportunity to engage and educate women on the importance of comprehensive dual protection strategies.

### Sexual Pleasure and Condom Use

For some CBW, decisions to not use condoms were associated with seeking pleasure, specifically addressing reduced sexual pleasure and physical discomfort when using condoms. These concerns are consistent with broader research showing that diminished pleasure is a key barrier to condom use for many women [21, 81, 82]. Individuals may avoid or be reluctant to use condoms due to the perception that condoms reduce sexual pleasure [82]. This barrier may be compounded by cultural and relational dynamics where sexual pleasure and intimacy are central to relationship satisfaction, yet condom use is perceived as disruptive to these experiences [18, 83, 84]. Ignoring pleasure as a factor associated with condom use decision making could undermine condom acceptability and consistent use, which in turn may increase vulnerability to acquiring HIV/STIs and unintended pregnancy. Integrating pleasure-affirming messaging with respect to condoms may help promote more consistent and empowered condom use. Tailored interventions that validate women’s sexual experiences and address both pleasure and practical barriers can improve HIV/STI prevention outcomes among CBW.

Some CBW’s displeasurable experiences with condoms may also reflect unmeasured aspects of condom use knowledge or application skills. Prior studies show that user error, including incorrect application, breakage, or slippage during vaginal and/or anal sex, is common and contributes substantially to condom failure [85–88]. Although this study did not assess condom use skills directly, intervention research demonstrates that building these skills enhances women’s self-efficacy to use condoms and support more consistent condom use, particularly among Black women [89, 90]. According to social cognitive theory, individuals with stronger condom use self-efficacy are more likely to initiate condom-related discussions with partners, exert greater effort to use condoms consistently, and persist in negotiating condom use despite a partner’s resistance [91]. Skill-building strategies, such as modeling correct condom use, practicing negotiation scenarios, or role-playing with feedback, strengthen self-efficacy, improve condom use outcomes, and have been central to interventions like Sister-to-Sister, which is a brief, culture-sensitive, gender appropriate HIV/STI prevention program that uses empowering “Respect Yourself! Protect Yourself! Because You Are Worth It!” messaging to strengthen condom use and negotiation skills [90, 92]. These patterns emphasize the importance of integrating condom use skill-building into HIV/STI prevention strategies for CBW alongside approaches that address pleasure, communication, and partner negotiation.

### Implications for Clinical Practice, Research, and Public Health

The findings from this study have implications for clinical practice, public health programming, and policy considerations. First, healthcare providers and public health practitioners must recognize that effective sexual health promotion requires addressing relationship dynamics alongside individual risk factors. The prevalence of relational context as a reason for condom non-use suggests that interventions should explicitly address trust and monogamy assumptions within established relationships. Couples-centered approaches towards HIV/STI prevention [77–79] that help couples understand the distinction between emotional trust and sexual health protection may be particularly valuable. Also, educating providers and couples about couples HIV testing and counseling (CHTC) may prove useful. CHTC is a risk reduction strategy where couples participate in the cycle of HIV/STI testing together (i.e., receipt of pretest information, counseling and risk assessment, results from the test(s), and post-test counseling [77, 79, 93–95]). Couples can also discuss their current HIV prevention strategies together, learn their HIV/STI status together, and develop a shared plan for maintaining their HIV/STI related health. Providers should be prepared to discuss how monogamy agreements, while emotionally meaningful, does not eliminate HIV/STI risk.

Second, the intersectional experiences of Black women in sexual health contexts deserve continued research attention. The interplay of factors influencing condom use decisions among CBW demands multifaceted approaches that move beyond traditional individual-level interventions. This study’s findings should be explored within broader frameworks that consider how racism, sexism, and other forms of systemic oppression shape sexual health opportunities and constraints, which can limit safer condom use [96, 97]. Previous literature indicates that due to racial and sex-based and gender biases, Black women may experience unique challenges in negotiating condom use due to experiences of racial and gendered oppression [97, 98] and may be more likely to conform to stereotypical racial and gender norms or be more passive in relationships [61] (i.e., not using a condom because their male partner(s) doesn’t want to). Importantly, the present findings emphasize that women should not bear sole responsibility for condom use within heterosexual relationships. Men’s attitudes, preferences, and coercive or dismissive behaviors substantially shape women’s ability to use condoms, underscoring the need for interventions that directly target heterosexual male partners as part of comprehensive HIV/STI prevention. Moreover, prior research recommends multi-level and multi-model integrated strategies for improving access to and engagement with contraceptive counseling that address social and structural determinants of health that maintain disparities in contraceptive use (e.g., condom use) for Black women [96, 99].

Third, this study’s findings highlight policy-level opportunities to support improved sexual health outcomes among CBW. Healthcare policy should support the colocation and integration of sexual health services with family planning and primary care to address the contraceptive substitution patterns observed in this study [100, 101]. Women who rely on non-barrier contraceptive methods need accessible opportunities to discuss HIV/STI prevention and dual protection strategies. Policy initiatives that promote comprehensive sexual health services, rather than fragmented approaches that separate pregnancy prevention from HIV/STI prevention, could simultaneously address the sexual and reproductive health needs revealed in this research.

### Limitations

The generalizability of these findings is limited as participants in this study resided in three metropolitan areas of Texas, which may not be representative of the broader population of CBW. Social desirability bias is another limitation, as participants may have responded in a manner they perceived to be socially acceptable, rather than providing entirely honest or reflective answers about their behaviors. However, participants were informed of the confidentiality of this study and reminded throughout the study survey that they could stop at any time and could skip questions they were uncomfortable answering. Additionally, given the cross-sectional nature of this study and the method of data collection, we did not delve deeply into participants’ detailed sexual experiences; decipher between the types of trust participants were referring to in relation to their sexual partner(s) (e.g., trust in their relationship partner, sexual trust, sexual distrust, or microsocial trust (i.e., formation of “paradigms of safety” in decisions about sexual relationships [66])); or delve deeper into the types of relationships women had (i.e., ethical non-monogamy), particularly among women who were single (e.g., nonromantic, romantic, transactional, etc.). This information could have provided more context for understanding their condom use behaviors. As such, focus groups or in-depth interviews might have provided more robust qualitative data about condom use behaviors and its relation to women’s HIV prevention strategies and family planning. The inability to conduct follow-up interviews specific to condom-use further limited the depth of information gathered.

Because the survey did not collect information on what sex was exchanged for, we were unable to interpret how different forms of exchange (e.g., monetary, housing, goods) may shape HIV vulnerability; this remains an important contextual gap. Prior research suggests that sex exchange often occurs within broader conditions of financial insecurity, housing instability, and limited socioeconomic opportunity, which are factors that significantly increase women’s HIV vulnerability [102]. The study also did not inquire about the number of sexual partners participants currently had, nor the exact frequency of sexual encounters in which condoms were not used during the past six months prior to study participation. A temporal approach would have allowed for a better understanding of condom use frequency and HIV/STI susceptibility within the sample. However, data collection for this study occurred during the COVID-19 pandemic, when many individuals were staying indoors, potentially impacting their condom use behaviors and access to condoms or other contraceptive methods during that time.

Our survey did not include a measure of participant’s (or knowledge about their partner’s) condom use knowledge or skills, which limited our ability to assess whether condom use errors, such as delayed application, early removal, or incorrect use, may have contributed to participants’ condom use experiences. This is another limitation given that past research shows that condom use errors are common among adults, and that user error, rather than product failure (a defective condom), accounts for a substantial proportion of condom failures (i.e., breakage, slippage, or premature removal during vaginal and/or anal sex) even among individuals who report consistent condom use [85–88]. These errors can significantly undermine the effectiveness of condoms in preventing HIV/STIs and increase the risk of HIV/STI exposure [85–88].

Future research should prioritize longitudinal study designs inclusive of qualitative methods that can capture the dynamic nature of condom use decisions across different relationship stages and life contexts. The cross-sectional nature of this study limits our understanding of how condom use patterns change as relationships evolve, individuals age, or life circumstances shift. Longitudinal research could illuminate critical transition points where interventions might be most effective and help identify factors that predict sustained protective behavior over time. Additionally, subsequent studies should incorporate validated measures of condom use knowledge and skills (e.g., the Condom-Use Skills Checklist) to clarify how these factors shape CBW’s condom use experiences and to distinguish skill-related challenges from those related to partner dynamics, trust, or contraceptive practices [103].

## Conclusion

The study’s findings reveal that CBW navigate complex reproductive and sexual health decision-making. Yet, persistent challenges around sexual communication and condom negotiation emphasize the continued need for interventions that strengthen women’s sexual health agency while also engaging partners as collaborative participants in sexual health protection. Notably, participants’ emphasis on pregnancy prevention in relation to STI prevention highlights a critical entry point for intervention. Leveraging existing engagement in family planning as a pathway to integrate and co-locate sexual and reproductive health services may offer a pragmatic and culturally grounded strategy for advancing STI and HIV prevention, inclusive of condom use. Moreover, strategies are needed to address relationship dynamics and broader social contexts that impact condom use decision making. By addressing individual, relational, and intrapersonal factors simultaneously, culturally responsive approaches can be developed to honor CBW’s experiences and support their sexual health goals within the context of their relationships.

## Figures and Tables

**Table 1:** Characteristics of study sample, by condom use behavior in the past 6 months (*N* = 154)

Demographics	Used condoms (*N* = 75)	Did not use condoms (*N* = 79)	Full study sample (*N* = 154)
	*N* (%)	*N* (%)	*N* (%)
Age [Range 19–70]: M (SD)	29.8 (8.29)	36.8 (11.6)	33.4 (10.7)
18–24	21 (28.0%)	6 (7.6%)	27 (17.5%)
25–34	42 (56.0%)	39 (49.4%)	81 (52.6%)
35–44	6 (8.0%)	16 (20.3%)	22 (14.3%)
45–54	4 (5.3%)	11 (13.9%)	15 (9.7%)
55+	2 (2.7%)	7 (8.9%)	9 (5.8%)
Ethnicity^b^			
Non-Hispanic	68 (90.7%)	76 (96.2%)	144 (93.5%)
Hispanic	4 (5.3%)	3 (3.8%)	7 (4.5%)
Employment^b^			
Unemployed	23 (30.7%)	17 (21.5%)	40 (26.0%)
Employed	51 (68.0%)	62 (78.5%)	113 (73.4%)
Education			
Less than a bachelor’s degree	28 (37.3%)	36 (45.6%)	64 (41.6%)
Bachelor’s degree or higher	47 (62.7%)	43 (54.4%)	90 (58.4%)
Income			
< $35k	19 (25.3%)	22 (27.8%)	41 (26.6%)
≥$35k	56 (74.7%)	57 (72.2%)	113 (73.4%)
Family planning provider			
No	37 (49.3%)	28 (35.4%)	65 (42.2%)
Yes	38 (50.7%)	51 (64.6%)	89 (57.8%)
Currently having sex with a male partner			
No	10 (13.3%)	6 (7.6%)	16 (10.4%)
Yes	65 (86.7%)	73 (92.4%)	138 (89.6%)
Ever used a form birth control			
No	22 (29.3%)	10 (12.7%)	32 (20.8%)
Yes	53 (70.7%)	69 (87.3%)	122 (79.2%)
Sexual orientation			
Heterosexual	66 (88.0%)	68 (86.1%)	134 (87.0%)
Other	9 (12.0%)	11 (13.9%)	20 (13.0%)
Relationship status			
Single	40 (53.3%)	16 (20.3%)	56 (36.4%)
In a relationship	35 (46.7%)	63 (79.7%)	98 (63.6%)
*Type of relationship* (*n*=98)			
Monogamous relationship (only have sex with one another and no one else)	29 (38.7%)	56 (70.9%)	85 (55.2%)
Monogamous relationship, but sometimes have sex with other people together as a couple (e.g.,	3 (4.0%)	5 (6.3%)	8 (5.2%)
threesome)			
Open relationship (allowed to have sex with other people separately and/or together)	1 (1.3%)	2 (2.5%)	3 (1.9%)
Other	1 (1.3%)	0 (0%)	1 (0.6%)
*Relationship length* (*n* = 98)			
< 1 year	10 (13.3%)	6 (7.6%)	16 (10.4%)
1–3 years	11 (14.7%)	21 (26.6%)	32 (20.8%)
> 3 years	14 (18.7%)	36 (45.6%)	50 (32.5%)
Personal HIV risk factors^c^	Used condoms (*N* = 75)	Did not use condoms (*N* = 79)	Study sample (*N* = 154)
Engagement in risk behavior(s) in past 6 months^a^			
Condomless vaginal sex with a male partner	74 (98.6%)	79 (100.0%)	153 (99.3%)
Condomless anal sex with a male partner	17 (22.6%)	9 (8.9%)	26 (17.1%)
Taken illicit drugs by needle injection	1 (1.3%)	0 (0.0%)	1 (0.6%)
Sex exchange	12 (16.0%)	0 (0.0%)	12 (7.8%)
Diagnosis of sexually transmitted infection (STI)	1 (1.3%)	3 (3.80%)	4 (2.6%)
Engagement in risk behaviors in past 6 months			
1 risk behavior only	51 (68.0%)	67 (84.8%)	118 (76.6%)
> 1 risk behavior	24 (32.0%)	12 (15.2%)	36 (23.4%)
Tested for STIs (other than HIV) at least once in their lifetime			
No	19 (24.1%)	31 (41.3%)	50 (32.5%)
Yes	60 (75.9%)	44 (58.7%)	104 (67.5%)
HIV testing history in the past 12 months (among those who tested at least once for an STI in their lifetime)			
Once	26 (32.9%)	7 (9.3%)	33 (21.4%)
> 1	21 (26.6%)	18 (24.0%)	39 (25.3%)
Not in the past 12 months	13 (16.5%)	19 (25.3%)	32 (20.8%)
Condom use behavior	Used condoms (*N* = 75)	Did not use condoms (*N* = 79)	Study sample (*N* = 154)
Frequency of condom use during vaginal sex in the past 6 months^b^			
Always	8 (10.7%)	0 (0%)	8 (5.2%)
Sometimes	67 (89.3%)	0 (0%)	67 (43.5%)
Never	0 (0%)	78 (98.7%)	78 (50.6%)
Ever discussed using condoms with a sexual partner(s)			
No	5 (6.7%)	23 (29.1%)	28 (18.2%)
Yes	70 (93.3%)	56 (70.9%)	126 (81.8%)
*First person to bring up the topic of using condoms* (*n* = 126)			
My partner	13 (17.3%)	7 (8.9%)	20 (13.0%)
Me	50 (66.7%)	33 (41.8%)	83 (53.9%)
I don’t remember	7 (9.3%)	16 (20.3%)	23 (14.9%)
*I wanted to use condoms* (*n* = 126)			
No	9 (12.0%)	15 (19.0%)	24 (15.6%)
Yes	61 (81.3%)	41 (51.9%)	102 (66.2%)
*My partner wanted to use condoms* (*n* = 110)			
Yes	36 (48.0%)	22 (27.8%)	58 (37.7%)
No	26 (34.7%)	26 (32.9%)	52 (33.8%)

a Total number of responses exceeds 154 as participant could select more than 1 response option (i.e., “All that apply”)

b Variables with missing data: ethnicity (*n* = 3), employment (*n* = 1), condom use frequency (*n* = 1)

c Variation in HIV risk behaviors across condom use groups should be interpreted in context, as differences may reflect underlying demographic or relational characteristics rather than condom use itself. Condom use categories were dichotomized (users vs. non-users) for descriptive purposes to align with the qualitative focus of the study

**Table 2:** Themes describing women’s use of condoms (*N* = 75)

Themes^a,b^	Definition of Themes	Example Quotes
Relational Context (*n* = 10)	The significance of familiarity with a sexual partner which influenced condom use decision-making	*Did not know my partner that well* *I was not sure about the status of the person I had indulged in sex with* *New partner* *Non-committed relationship* *Someone I didn’t know*
HIV/STI Prevention (*n* = 39)	Minimizing acquisition of sexually transmitted infections (STIs) and HIV through condom use	*HIV Protection* *STD prevention* *Protecting myself from being infected with sexually transmitted disease*. *Protection from STDs and HIV Safe sex* *When I had sex for the first time, we forgot to put a condom on. The second time we had sex, we used a condom because we forgot the first time and were taught to have safe sex*
Family Planning (*n* = 36)	The use of contraceptive methods to prevent unintended pregnancies	*Former partner and I were mutually exclusive, so we primarily used condoms to prevent pregnancy* *I was in my ovulation day, and we were not planning to become pregnant anytime soon* *To reduce risk of getting pregnancy and contracting STD since I had sex with someone who was not my partner* *To avoid getting pregnant*, *especially when it was my ovulation day. It’s much safer to use a condom rather than a birth control pill*
Contextual Factors (*n* = 6)	Condom use influenced by personal preference and availability of condoms	*Personal decision not to They were available*

Notes.

a Parentheses represent the number of participants that provided a text comment related to the associated theme

b Some participants addressed their condom use within the context of more than one theme. When coding, we separated text responses and categorized the phrases based on the teams assumed and discussed context

**Table 3:** Themes describing women’s decisions to not use condoms (*N* = 79)

Themes^a,b^	Definition of Themes	Example Quotes
Relational Context (*n* = 65)	The significance of relational context with a sexual partner, inclusive of relationship type, dynamics, and length, as well as familiarity and partner’s preferences, which influenced women’s sexual decision-making	*We are married and we trust each other* *We are exclusive* *We are in a relationship where we only have sex with each other only* *Committed relationship* *We both agreed to be monogamous* *He is my partner* *We trust each other* *Trust; been with same man for 6 years* *Same partner × 30 years* *Familiar with partner* *Partner does not like them* *My partner didn’t want to use* *Partner desire*
HIV/STI Prevention (*n* = 3)	Minimizing acquisition of sexually transmitted infections (STIs) and HIV through safer sex practices: HIV/STI testing	*Both test for STD and HIV regularly together* *We both have been tested*
Family Planning (*n* = 10)	The use of contraceptive methods to prevent unintended pregnancies, or the decision not to use condoms due to planning to conceive or being pregnant	*I’m on birth control* *We talked about family planning* *We are currently expecting* *I’ve had a tubal ligation* *My tubes are tied*
Intrapersonal Factors (*n* = 22)	Women’s personal attitudes toward condom use, including concerns that condoms may lessen sexual pleasure or physical comfort with their male sexual partner(s)	*Pleasure* *I did not want to feel a condom* *They’re uncomfortable* *I don’t like them* *At times I could be so drunk that we forget to use a condom, but I always ensure* *I take a birth control pill afterwards*. *I do not enjoy them* *Just for fun* *Forgot*

a Parentheses represent the number of participants that provided a text comment related to the associated theme

b Some participants addressed their condom use within the context of more than one theme. When coding, we separated text responses and categorized the phrases based on the teams assumed and discussed context

## Data Availability

The data that support the findings of this study are available from the corresponding author upon reasonable request.

## Code Availability

The data that support the findings of this study are available from the corresponding author upon reasonable request.
